# IUC24408-83 Outcomes of distal ureterectomy with boari flap reconstruction for ureteric transitional cell carcinoma

**DOI:** 10.1093/oncolo/oyaf248.020

**Published:** 2025-08-29

**Authors:** R Abdlbagi, S Ahmed, H Nour, A Abdelrahmn, T Chang, H Motiwala

**Affiliations:** Southend Hospital, United Kingdom; Southend Hospital, United Kingdom; Southend Hospital, United Kingdom; Southend Hospital, United Kingdom; Southend Hospital, United Kingdom; Southend Hospital, United Kingdom

## Abstract

**Background:**

To assess functional outcomes, study the suitability of distal ureterectomy in the management of distal ureteric TCC in terms of risk of recurrence and complications with or without psoas hitch or Boari flap in the reconstruction and repair of the ureter. Upper tract urothelial carcinomas (UTUC) account for only 5%-10% of UCs with an estimated annual incidence in Western countries of almost 2 cases per 100,000 inhabitants (1). In addition, approximately two-thirds of patients who present with UTUCs have muscle-invasive disease at diagnosis compared to 15%-25% of patients diagnosed with *de novo* BC, and this is the reason that makes radical nephroureterectomy the logical Gold standard treatment (2). Moreover, kidney-sparing surgery for low-risk UTUC reduces the morbidity associated with RNU (eg, loss of kidney function), without compromising oncological outcomes (3). We present a series of 23 patients who were managed with open and robotic distal ureterectomy and baori flap reconstruction.

**Methods:**

Data were collected retrospectively on 2 consultants’ patients, operated on between 2020 and 2024.

**Results:**

We treated 23 patients with a mean age of 75 years (47-91 years). There were 19 males and 4 females. Twelve were on the right and twelve on the left, and 2 cases were done via robotic assistance. The mean follow-up was 34 months (2.8 years) (10-58 months), with 2 upper tract recurrences at the distal ureter, and 6 patients developed non-muscle invasive Bladder TCC. In this cohort, 5 patients passed away, and one of them, post-palliative distal ureterectomy, revealed a local advanced distal ureteric mass.

**Conclusions:**

In selected cases, distal ureterectomy and baori flap may be considered, as it has an acceptable functional outcome and severe postoperative complications were rare, with a relatively low risk of recurrence in the short term.

